# *Paeoniflorin* has anti-inflammation and neurogenesis functions through nicotinic acetylcholine receptors in cerebral ischemia-reperfusion injury rats

**DOI:** 10.22038/IJBMS.2018.30371.7322

**Published:** 2018-11

**Authors:** Cheng-Hang Ko, Chun-Ping Huang, Yi-Wen Lin, Ching-Liang Hsieh

**Affiliations:** 1 Department of Chinese Medicine, China Medical University Hospital, Taichung 40447, Taiwan; 2 Chinese Medicine Research Center, China Medical University, Taichung 40402, Taiwan; 3 Graduate Institute of Acupuncture Science, College of Chinese Medicine, China Medical University, Taichung 40402, Taiwan; 4 Graduate Institute of Integrated Medicine, College of Chinese Medicine, China Medical University, Taichung 40402, Taiwan

**Keywords:** Apoptosis, Inflammation, Neurogenesis, Nicotinic acetylcholine – receptor, Paeoniflorin, Stroke

## Abstract

**Objective(s):**

*Paeoniflorin* (PF) has anti-oxidation, anti-inflammation, anti-apoptosis, and neuroprotection pharmacological effects against ischemic injury. The aim of the present study was to investigate the neuroprotection mechanisms of PF in cerebral ischemia-reperfusion injury rats.

**Materials and Methods:**

We established an animal model of cerebral infarct by occlusion of the middle cerebral artery for 15 min, followed by reperfusion, and PF was administered 24 hr later (20 mg/kg, intraperitoneally for 6 days) after reperfusion

**Results:**

Treatment with PF reduced the neurological deficit score, improved motor function, decreased cell counts of nicotinic acetylcholine receptor (nAChR) α4β2 immunoreactive cells, and increased cell counts of nAChR α7. Furthermore, PF administration suppressed neuronal apoptosis and promoted neurogenesis.

**Conclusion:**

PF rescued neurological deficit and underlying mechanisms were inhibition of neurological apoptosis and inflammation by nAChRs.

## Introduction

Stroke is the second common cause of death and morbidity in developed nations. Ischemic stroke causes neural cell damage or death, it further induces an inflammatory response to eliminate debris. However, excessive post-stroke inflammatory responses limit brain tissue recovery and neurogenesis. Anti-inflammatory strategies have potential for therapy in stroke (1). Numerous traditional medicinal herbs, such as PF, have effects of anti-oxidation, anti-inflammation, anti-apoptosis, and neuroprotection in experimental animal models (2). PF is the principal component in *Paeonia lactiflora* Pall. and has been reported to inhibit inflammation (3, 4), have neuroprotective effects (5), and mediate cerebral infarct preconditioning by comparative proteomics analysis (6).

Phagocytic myeloid cells play an important role in ischemic tissue repair and re-organization, but may also amplify the inflammatory response to induce stronger secondary tissue damage. Anti-inflammation therapy should be involved, notably, nAChRs protects neurons in neurodegeneration (7), ischemia (8), and intracerebral hemorrhage (9). The most abundant subtypes of nAChRs in the brain are heteromeric α4β2 and homomeric α7 (10). nAChRs α4β2 is an inflammatory marker of cerebral ischemia (11), otherwise, nAChR α7 modulators enhance both enhance synaptic plasticity (12) and neurogenesis (13).

Our previous study found that PF reduced the ratio of cerebral infarction area and counts of inflammatory cells. In this study, we hypothesize that PF arose anti-inflammation and neurogenesis by activation of nAChRs and further investigated the effects of PF administration by a neurological status test and immunohistochemical staining. Our data demonstrated that PF could reduce the neurological deficit score and the counts of α4β2 cells, increase both nAChR α7 and Ki-67 (mitotic cell marker) immunoreactive cells; it suggested PF may be an effective therapy option for stroke.

## Materials and Methods

***Animals***

The total of eighteen male Sprague Dawley (SD) rats weighing 250–350 g were used in this study and purchased from BioLASCO (Taipei, Taiwan). A 12:12 hr light-dark cycle was maintained, the relative humidity was controlled at 55±5%, and the room temperature was controlled at 25±1 ^°^C. Adequate food and water were provided. The Animal Care and Use Committee of China Medical University approved the use of these animals. In addition, all procedures were performed according to the Guide for the Use of Laboratory Animals (National Academy Press).

***Establishment of ischemia-reperfusion injury animal model***

Cerebral ischemia was induced by intraluminal suture occlusion of the middle cerebral artery (MCA), as described previously (14). The rats were anesthetized with isoflurane (Baxter, Guayama, Puerto Rico) by using a vaporizing system (MATRX VIP 3000; Midmark, USA) and then placed on a stereotaxic apparatus in the prone position. The parietal bone was thinned using a grinding machine to monitor the blood flow. A Laser Doppler flowmetry probe (DRT4; Moor Instrument Ltd., England) was placed 2.5 mm lateral, 2 mm posterior to the bregma to measure the blood flow of MCA. We set Laser Doppler flowmetry values as a monitor during the surgery process and values of blood flow before surgery were more than 500 min/div. The rats were then placed in the supine position, and the neck was incised from the midline to expose the right common carotid artery. The right internal and common carotid arteries were clipped, the external carotid artery was permanently ligated, and an incision was made. A 3-0 nylon filament suture, the tip of which was smoothed by heat and coated with poly-L-lysine (UNIK, Taiwan), was advanced for a distance of approximately 20 mm from the incision of the external carotid artery through the common carotid artery into the internal carotid artery to block the origin of the MCA. The value of the MCA was declined below100 min/div and blockage of cerebral blood flow was confirmed. In the present study, the cerebral blood flow of the right MCA was blocked for 15 min and then restored.

***Grouping***

The rats were randomly divided into three groups as follows: (1) sham group (SG): the common carotid artery of rats was exposed without blocking the cerebral blood flow of the MCA; (2) control group (CG): the cerebral blood flow of the right MCA was blocked in order to induce ischemia-reperfusion injury; (3) PF group (PG): PF (Tauto Biotech, Shanghai, P.R.C.) was administered (20 mg/kg, dissolved in glycofurol solution and further dilution with phosphated buffer saline) intraperitoneally 24 hr after surgery for 6 days.

***Neurological status evaluation***

The neurological status was evaluated by a staff blinded to the groups according to the Modified Neurological Severity Score at 24 hr before and after surgery as described previously (14). In brief, total neurological deficit score is 18 and comprises motor test (raising the rat by the tail, 0–3; placing the rat on the floor, 0–3), sensory test (0–2), beam balance test (0–6), and reflex test (0–4) scores.

***Rotarod test***

The rats were placed on a Rotamex (Columbus Instrument, Ohio, USA) with an initial speed of 4 rpm, which increased by 1 rpm every 8 sec until the maximum speed of 40 rpm was attained at day 7 after surgery. The latency spent by the rat on the rotarod before stepping out was recorded, the test was performed five times, and the average of the three longest times recorded was calculated.

***Immunohistochemical staining***

The rats were further anesthetized with overdose of chloral hydrate, perfused with 0.9% saline, and then brains were removed. The brains were fixed in 4% paraformaldehyde for 3 days and transferred to 30% sucrose (w/v) for 4 days. The brains were embedded in optimal cutting temperature (OCT) medium (Leica Surgipath, USA) and cut into 20 μm sections in a cryostat (Leica, USA), rinsed with 0.01% Tween 20 / phosphate buffered saline (PBS-T) twice and soaked in 3% H_2_O_2_/methanol for 15 min to inhibit endogenous peroxidase activity. The sections were then blocked with 10% normal goat serum (Genemed Biotechnologies, CA, USA) for 20 min at room temperature. The sections were incubated with a primary antibody, nAChRs α4β2 or Ki67 (1:200) (Millipore, Washington, DC, USA), at 4 °C overnight in a moisture chamber. The sections were subsequently incubated with the biotinylated-conjugated secondary antibody (Genemed Biotechnologies, CA, USA) for 10 min at room temperature, followed by incubation with the streptavidin-peroxidase complex (Genemed Biotechnologies, CA, USA). The sections were visualized using 3,3’-diaminobenzidine (Scytek Laboratories, UT, USA) as the chromogen and counterstained with hematoxylin (Genemed Biotechnologies, CA, USA). During the incubation steps, the sections were washed with PBS-T three times for 10 min per cycle. The stained sections were mounted in a mounting media (Assistant-Histokitt, Germany), and the photographs were captured under a microscope (Olympus, BX-51, Japan).

***Immunofluorescence staining***

The sections were prepared as above described and blocked with 10% normal goat serum (Jackson ImmunoResearch Laboratories, West Grove, PA, USA) for 20 min at room temperature. The sections were incubated with a primary antibody, CD68 (1:200) (Millipore, Washington, DC, USA) and nAChR α7 (1:200) (Alomone, Jerusalem, Israel) at 4 °C overnight. The secondary antibodies were incubated with Goat anti-mouse IgG DyLight488 (1:1000, Jackson ImmunoResearch Laboratories, West Grove, PA, USA) and Goat anti-rabbit IgG DyLight594 (1:1000, Jackson ImmunoResearch Laboratories, West Grove, PA) for 2 hr at 4 °C. The stained sections were mounted with DAPI (Sigma, St. Louis, MO, USA), sealed under a coverslip, and then photographed using a fluorescent microscope system (Olympus, BX-51, Japan).

***TUNEL assay***

The brain sections were prepared as above described and soaked in 3% H_2_O_2_/methanol for 15 min to inhibit endogenous peroxidase activity. Apoptotic cells were evaluated using the FragEL DNA fragmentation Detection kit (Calbiochem), following the manufacturer’s protocol.

***Statistical analysis***

All data were presented as mean ± standard deviation. Statistical significance was analyzed through one-way ANOVA, followed by Tukey’s *post hoc *test. A *P-*value of <0.05 was considered statistically significant.

## Results

***Effect of Paeoniflorin on neurological deficit score in rats with cerebral ischemia-reperfusion injury***

A cerebral infarct animal model was established through vascular occlusion and confirmed by Laser Doppler flowmetry. The neurological deficit score test was examined at days 1 and 7 after surgery. PF was administered intraperitoneally at 24 hr after surgery for 6 days. The neurological deficit scores in the CG and the PG groups were higher compared to SG at day 7 after reperfusion (both *P<0.05, Table 1). The neurological deficit scores of PG were significantly reduced compared to CG at day 7 (^#^*P<* 0.05, Table 1).

**Figure 1:**
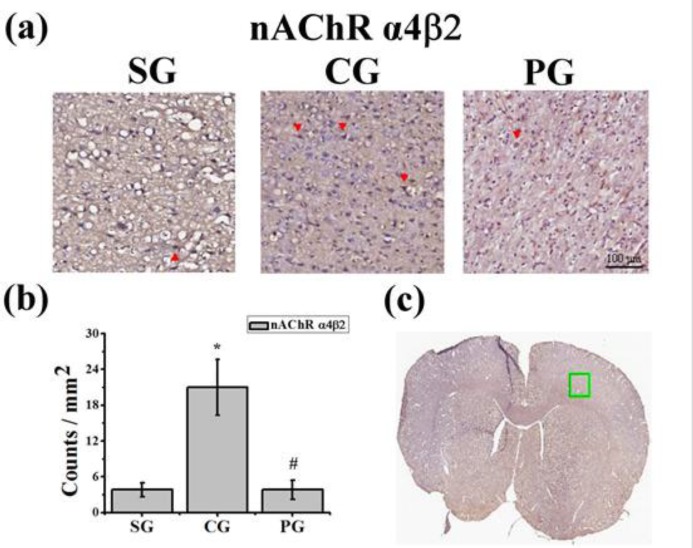
The immunohistochemical staining of nicotinic acetylcholine receptors α4β2 in the third total brain coronal section from the frontal lobe. (a) The nAChR α4β2 immunoreactive cells were marked by arrowhead (200X, scale bar = 100 μm). (b) The counts of nAChR α4β2 immunoreactive cells were increased in the CG group compared to the SG group and reduced in the PG group compared to the CG group. *P<0.05 compared to the SG group. #*P*<0.05 compared to the CG group. (c) The immunoreactive cells were counted manually in the third total brain coronal section from the frontal lobe as shown (green square, 1 x 1 mm2). SG: sham group; CG: control group; PG: Paeoniflorin group

**Figure 2:**
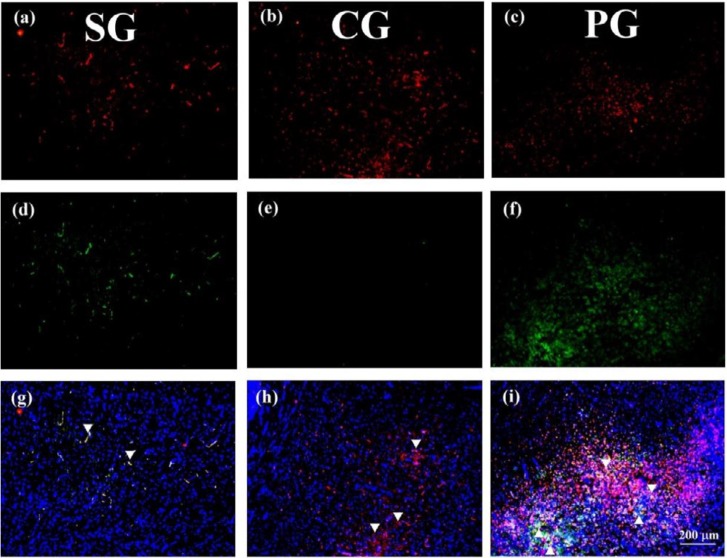
The immunofluorescence staining of CD68 and nicotinic acetylcholine receptor α7. The CD68 (red, a-c), nAChR α7 (green, d-f) immunoreactive cells were merged with DAPI (g-i) and co-expression cells were marked by arrowhead (100X, scale bar = 200 μm, three independent experiments)

**Figure 3:**
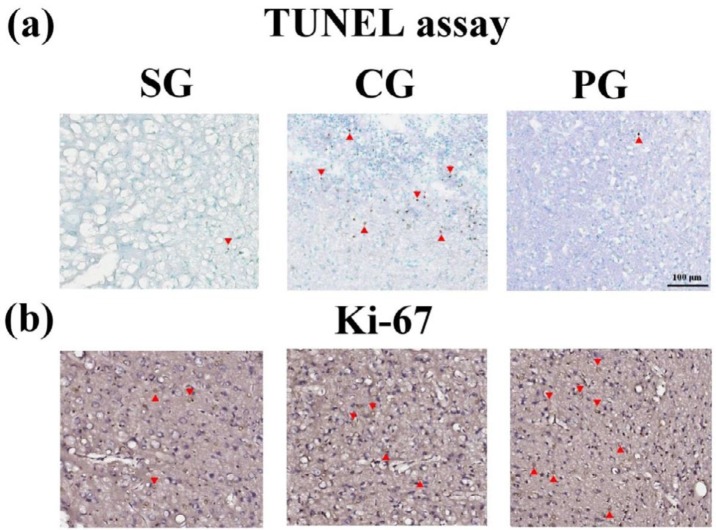
The immunohistochemical staining of the TUNEL assay and Ki-67 immunoreactive cells. (a) Apoptotic cells and (b) The Ki-67 immunoreactive cells were marked by arrowhead (200X, scale bar = 100 μm)

**Table 1:** Neurological deficit score test

Group	Day 1 after surgery	Day 7 after surgery
SG	0.00 ± 0.00	0.00 ± 0.00
CG	7.30 ± 0.47	7.67 ± 0.47 *
PG	7.00 ± 0.00	5.67 ± 0.94 *#

Eighteen male Sprague-Dawley rats were randomly divided into three groups and neurological deficit score test was evaluated by a well-trained staff blinded to the groups according to the Modified Neurological Severity Score at 1 and 7 days after surgery. Data were represented as mean ± SD.

* *P<*0.05 compared to the SG group.

# *P<*0.05 compared to the CG group. Statistical comparisons were performed by one-way ANOVA with Tukey-Kramer*** post hoc*** test.

**Table 2:** Latency to step out in the rotarod test

Group	Latency **(sec)**
SG	154 ± 42.3
CG	53.9 ± 29.3
PG	121.5 ± 13.8#

The rats were placed on a rotarod and time of latency to step out was recorded **(sec)** at day 7 after neurological status evaluation test. Data were represented as mean ± SD.

# *P<*0.05 compared to the CG group.

**Table 3:** The counts of Ki-67 and TUNEL assay reaction-positive cells

Group	TUNEL assay (+)	Ki-67 (+)
SG	3.6 ±1.9	16.8 ± 10.1
CG	42.8 ± 45.5	51.0 ± 30.6
PG	3.0 ±1.9#	96.5 ± 62.0#

The apoptotic cells were reduced in the PG group compared to the CG group, otherwise Ki-67 immunoreactive cells were increased in the PG group compared to CG. Data were represented as mean ± SD.

# *P<*0.05 compared to the CG group.

***Effect of Paeoniflorin on rotarod test in rats with cerebral ischemia-reperfusion injury***

We further estimated the motor function of rats by rotarod test at day 7 after the neurological deficit score test. The latency of the CG was 53.9 ± 29.3 sec and was significantly increased in the PG (121.5 ± 13.8, ^#^*P<* 0.05, Table 2).

***Administration of Paeoniflorin reduced the counts of nAChR α4***β***2 immunoreactive cells***

To underlay the mechanism of PF for ischemic tissue repairing, we examined nAChR α4β2 by using immunohistochemical staining (Figure 1a) in the third total brain coronal section from the frontal lobe (Figure 1c). The counts of nAChR α4β2 immunoreactive cells were significantly increased in the CG group (21.0±4.6, *P<0.05, Figure 1b) compared to the SG group (3.8 ± 1.1, Figure 1b), and reduced in the PG group (3.8±1.6, #*P<* 0.05, Figure 1b) compared to the CG group.

***Administration of Paeoniflorin raised the expression of*** ***nicotinic acetylcholine receptors ***α7 microglia

We further examined microglia marker CD68 (red) and nAChR α7 (green). There was normal distribution of nAChR α7 microglia in the SG group (Figure 2g, marked by arrowhead), but decreased distribution in the CG group (Figure 2e). We observed the counts of CD68 immunoreactive microglia were increased (Figure 2 hr, marked by arrowhead). Notably, both of CD68 and nAChR α7 immunoreactive microglia were raised (Figure 2i, marked by arrowhead).

***Administration of Paeoniflorin downregulated apoptosis and increased the counts of the Ki-67 (+) cells***

The counts of the apoptotic cells were significantly decreased in the PG group compared to the CG group (3.0 ±1.9 vs. 42.8 ± 45.5, ^#^*P<*0.05, Figure 3a and Table 3). Notably, the counts of the Ki-67 immunoreactive cells were significantly increased in the PG group compared to the CG group (96.5 ± 62.0 vs. 51.0 ± 30.6,^ #^*P<*0.05, Figure 3b and Table 3).

## Discussion

In this study, we focused on the treatment of post-stroke; PF was administrated at 24 hr after surgery. Our data showed PF could improve the neurological deficit score and motor function (Tables 1 and 2). It suggested that PF administration improved the neuropsychological conditions after cerebral ischemia-reperfusion injury. Ischemic stroke causes hypoxia and results in metabolic failure leading to uncontrolled cell death. Ischemic inflammation facilitates the clearance of necrotic cells and debris. However, excess post-stroke inflammation hampers effective nerve cell repair (15, 16). Post-stroke inflammation reactions should be self-limited and resolved by inhibitory molecules within the immune system. Our data showed PF administration reduced nAChR α4β2 expressing cells and ameliorated the inflammatory response in the frontal lobe cortex (Figure 1).

Recent studies indicate the autonomic nervous system is involved in controlling inflammation via neural circuits to affect immune cells (17-19). One of these brain-immune connections is the nAChR α7 anti-inflammatory pathway derived through sensing pro-inflammatory cytokines by vagus nerves (20). The activation of the nAChR α7 has been demonstrated to improve functional recovery in ischemic stroke (21). Our previous study showed that pre-treatment of PF reduced pro-inflammatory cytokine releasing and apoptosis (4). In the present study, we further indicated PF activated the anti-inflammatory pathway through increasing the counts of nAChR α7 microglia and inducing a cholinergic anti-inflammatory pathway (Figure 2).

The nAChR α7 subtype has both anti-apoptotic effects through phosphatidylinositol 3-kinase (PI3K) (22) and proliferative effects through β-arrestin-mediated activation of Src pathways (23). We showed that PF administration decreased the numbers of apoptotic cells and promoted neurogenesis under post-stroke inflammation (Figure 3 and Table 3). It demonstrated that PF administration not only suppressed apoptosis but also promoted neurogenesis in cerebral ischemia-reperfusion injury rats. These effects may be caused by activation of nAChR α7 in the neuronal cells and non-neuronal cells within the nervous system.

## Conclusion

In summary, PF improves the neurological deficit score and motor function through inhibition of neurological apoptosis, and inflammation by nAChRs further promoted neurogenesis in the post-stroke rats. Our data suggested that PF may be an effective therapy option for post-stroke.
